# Smoking cessation programmes using traditional medicine in Korea

**DOI:** 10.1186/s12906-016-1462-9

**Published:** 2016-12-01

**Authors:** Soobin Jang, Yu Lee Park, Ju Ah Lee, Kyeong Han Kim, Eun-Kyoung Lee, Seung-Ho Sun, Yong-Cheol Shin, Seong-Gyu Ko, Sunju Park

**Affiliations:** 1 Department of Preventive Medicine, College of Korean Medicine, Kyung Hee University, 26 Kyungheedae-ro, Dongdaemun-gu, Seoul, 02447 Republic of Korea; 2 KM Fundamental Research Division, Korea Institute of Oriental Medicine, 1672 Yuseongdae-ro, Yuseong-gu, Daejeon, 34054 Republic of Korea; 3 Department of Korean Internal Medicine, College of Korean Medicine, Sang-ji University Korean medicine Hospital, 80 Sangjidae-gil, Wonju, 26338 Republic of Korea; 4 Department of Preventive Medicine, College of Korean Medicine, Daejeon University, 62 Daehak-ro, Daejeon, 34520 Republic of Korea

**Keywords:** Smoking cessation, Tobacco control, Traditional Korean medicine, Ear acupuncture

## Abstract

**Background:**

There are growing interests in using various methods including traditional and complementary medicines (T&CM) for tobacco control. The study aimed to introduce how traditional Korean medicine (TKM) applied to smoking cessation programmes in Korea and to show the detail information of each programme for designing other smoke cessation programmes.

**Methods:**

Reports of the smoke cessation programmes in Korea were searched on March 10th, 2016, from the webpages of the related agencies and the databases: the Ministry of Health and Welfare, the Korea Health Foundation, the Association of Korean Medicine, PubMed, Google scholar, the RISS, the KISS, the NDSL, and the OASIS. Smoking cessation programmes, projects, or services using traditional Korean medicine (TKM) were included with no language, implementation site, and year restrictions.

**Results:**

The three smoking cessation programmes using TKM in South Korea were the public health centre smoking cessation programme (PHC-SCP), the Ministry of Gender Equality & Family smoking cessation programme (MOGEF-SCP), and the National Health Insurance Service smoking cessation treatment project (NHIS-SCP). All programmes included ear acupuncture and counselling. Manual acupuncture was only used in the NHIS-SCP. The MOGEF-SCP and the NHIS-SCP used herbal medicines selectively. The PHC-SCP and MOGEF-SCP provided education programme and other tools such as non-smoking doll, self-writing handbook. They were run at no cost for participants. Treatment period were different for each programmes, 3 weeks, 4 weeks, 8 to 12 weeks, respectively. Treatment frequency was twice a week for PHC-SCP and MOGEF-SCP, and dependent on each clinic for NHIS-SCP.

**Conclusions:**

This study showed the summaries of the smoking cessation programme that used TKM. The three programmes and the detail information will be a reference for other countries that are going to apply T&CM to their smoking cessation programme. Though TKM integrated smoking cessation programmes had been contributed to stop smoking, persistent efforts are needed to develop more effective and various treatments. In addition, this study suggests that consistent support and systematic reporting system are needed to be successful in non-smoking strategy.

**Electronic supplementary material:**

The online version of this article (doi:10.1186/s12906-016-1462-9) contains supplementary material, which is available to authorized users.

## Background

Smoking is one of the biggest causes of death, and it is related to the death of one in ten people (6 million) every year. Smoking related death is expected to increase to 8 million by 2030 if no proper smoking cessation policies are implemented [1]. According to the World Health Organization (WHO), 21% of the world populations above 15 years old were smokers in 2012 [2]. To solve the problem, the WHO developed the Framework Convention on Tobacco Control (FCTC) in 2003, which is the first international treaty on public health regarding the reduction of smoking related diseases and death. The diagnosis and treatment of tobacco-dependence is included in the public health systems of 81 countries, and 72 of these systems include insurance that covers the cost of drugs for tobacco-dependence [3].

The most popular treatments of tobacco control are nicotine replacement therapy (NRT), varenicline, bupropion, cytisine, clonidine, nortriptyline, and escitalopram [4, 5]. Since most smokers are dependent on nicotine, it is difficult to be successful at smoking cessation through drug therapy alone [6]. Therefore, a comprehensive approaches including emotional support should be provided to smokers [7]. As adverse events from pharmaceuticals and NRT, such as sleep disorder, nausea, vomiting, and skin irritation, are reported, the development of a safer intervention is needed for smoking cessation [4, 5]. The success rate of smoking cessation can be increased when multiple methods are combined, such as counselling, education, complementary therapy, and regulatory policy [6].

There has been a growing interest in traditional and complementary medicine (T&CM) for smoking cessation treatment in many countries [8]. Traditional Korean medicine (TKM) is one of the T&CM that have been widely used in Korea and it has been a role in smoking cessation treatment along with western medicines and NRT for decades within the national health system of South Korea. Also, there have been many clinical trials or experimental studies about smoking cessation with acupuncture [9, 10]. In South Korea, tobacco control is included in the Health Plan 2020, and its goal is to reduce the smoking rate to 29% in men and to 6% in women by 2020 [11].

The objective of this study was to describe how TKM has been applied in smoking cessation programmes and how this can be expanded and used to improve other smoking cessation programmes that will increase smoke cessation rates. This study is the first research result of our STOP (Stop Tobacco Programme using TKM) study series.

## Methods

### Search strategy

Official reports about public health programmes were searched from the webpages of the government and related bodies: the Ministry of Health and Welfare (MW, www.mohw.go.kr), the Korea Health Foundation (www.khealth.or.kr), and the Association of Korean Medicine (AKOM, www.akom.org). Other information about the smoking cessation programmes was retrieved from the following databases: PubMed, Google, the RISS (Research Information Service System, http://www.riss.kr), the KISS (Korean Studies Information Service, http://kiss.kstudy.com), the NDSL (National Digital Science Library, http://www.ndsl.kr), and the OASIS (Korean Medicine Information System, https://oasis.kiom.re.kr). Additionally, a summary of and detailed information about the smoking cessation programmes or projects of TKM were investigated through the following smoking cessation information database systems and treatment guidelines: the TKM Public Service Report [12], the Public Health Information System (PHIS) [13], the Ministry of Gender Equality and Family (MOGEF) internal data [14], and the Guideline on Health Insurance supported smoking cessation treatment [15]. The first three reports were non-public, private reports and the last guideline can be found at www.nosmokeguide.or.kr. Additional data were obtained by directly contacting related institutions.

The data were searched for with the following keyword combinations on March 10th, 2016: “smok*”, “tobacco”, “nicotine”, and “cessation”, “stop”, “free”, “control”, “manage”, “treatment” and “program”, project” and “Korean medicine”, “integrative medicine”, “complementary and alternative medicine”, “acupuncture”, “ear-acupuncture”, “herbal medicine”, “herb”, “aromatherapy, “aroma”, “moxibustion”. All smoking cessation programs, projects, or services using TKM were included. There were no restrictions on language, implementation site and year.

### Data analysis

Two researchers searched and selected the studies and reports independently. When a disagreement occurred, a third researcher resolved. The data were extracted into the predefined data extraction form. General information about each project, such as the programme or project title, government agency, start year, programme conducting site, targeted population, budget, number of participants, and detailed contents of each programme were descriptively presented.

## Results

Smoking cessation programmes using TKM can be categorized into 3 groups: (1) public health centre smoking cessation programmes, (2) Ministry of Gender Equality & Family smoking cessation programmes focused on the teenagers, and (3) National Health Insurance Service smoking cessation treatment projects. The summaries of these 3 programmes are shown in Table 1. Detailed information regarding each programme is described in Table 2.

**Table 1:** Summary on TKM smoking cessation programmes in South Korea

TKM smoking cessation programme	PHC-SCP	MOGEF-SCP		NHIS-SCP
Targeted population	Teenagers, Adults	Teenagers		Anyone
Related agency	PHC, MW	MOGEF, MW		NHIS, MW
Start year	2001	2003		2015
Subtype	NA	Designation clinic system	Designation doctor system	NA
Conducting site	PHCs, Schools, Companies	TKM clinics	Schools	TKM clinics
Medical cost	No medical cost for the patients	No medical cost for the patients		Supports 80% of medical costs (not including TKM treatments)
Budget (per year)	NR	USD 50,000^a^		USD 87,000,000^a^
Number of participating institutions	9 PHCs (2001) →137 PHCs (2003) →35 PHCs (2007) →34 PHCs (2013)	908 TKM clinics (in 2014)	84 TKM doctors (in 2014)	1,270 TKM clinics (in March 2016)
Number of participants	238,951 (2005–2014)	96,003 (2003–2014)		162,010 (2015-present)
Evaluation index	Satisfaction, Cessation success rate	Cessation success rate		Cessation success rate
Characteristics	Oldest PHC-based programme typically integrated	Free programme for teenagers (a vulnerable group)		Clinic-based programme supported by the health insurance project fund

^a^ *USD* United States dollar. It was based on the annual average exchange rate in 2015 from the Korea Exchange Bank

*PHC-SCP* Public health centre smoking cessation program, *MOGEF-SCP* Ministry of Gender Equality & Family smoking cessation program, *NHIS-SCP* National Health Insurance Service smoking cessation treatment project, *TKM* Tradition Korean Medicine, *MW* Ministry of Health & Welfare, *NHIS* National Health Insurance Service, *PHC* Public Health Centre, *MOGEF* Ministry of Gender Equality & Family, *NA* not applicable, *NR* not reported

**Table 2:** Details of programmes using TKM for smoking cessation

Interventions		Program	PHC-SCP	MOGEF-SCP	NHIS-SCP
Korean medicine	Ear acupuncture	Needle type	Intradermal needle	Intradermal needle	Intradermal needle
		Acupoints	Lung, Shinmun, Endocrine, Pharynx & Larynx and Inner nose	Lung, Shinmun, Endocrine, Pharynx & Larynx and Inner nose	Lung, Shinmun, Endocrine, Pharynx & Larynx and Inner nose
		Duration	3–4 h	3–4 h	3–4 h
	Manual acupuncture	Needle type	NA	NA	Basic needle
		Acupoints	NA	NA	HT7, ST36, LI4, LU7, LU6
		Duration	NA	NA	15–20 min
	Herbal medicine		NA	Non-smoking pill (Optionally)	· Anxiety: Modified Xiaoyao Powder · Cough: Shensuyin · Phlegm: Banxia-Houpo-tang or Erchen-tang (Optionally)
Adjuvant therapy	NRTs		·Nicotine patch ·Nicotine gum	NA	· Nicotine patch · Nicotine gum
	Other tools		Non-smoking doll (showing harms of cigarettes)	Self-writing handbook	NA
	Consultation	Consultant	Health care provider	TKM doctor	TKM doctor
		Type	5A type counselling^a^	5A type counselling (Dependent on doctor)	5A type counselling (Dependent on doctor)
		Duration	10–15 min	5–10 min	5–10 min
	Education program		·Watching a video on harms of smoking ·Smoking detox experiment (goldfish experiment with cigarettes)	Watching a video on harms of smoking	NA
Basic information	Medical cost	Patient cost sharing	Free	Free	20%
		Government supports	100% support	100% support	80% support
	Treatment period		Dependent on each PHC (Typically 4 weeks)	3 weeks	8 or 12 weeks (Dependent on patient’s choice)
	Treatment frequency		Dependent on each PHC (Typically twice a week)	Twice a week	Dependent on each clinic
	Primary outcome		Exhaled CO	Self-reported cessation success rate	Self-reported cessation success rate
	Characteristics		Utilises a variety of methods for cessation	Mainly acupuncture and additional education	Similar to the course of treatment in TKM clinics

^a^5A type counselling: 5A means ‘Ask’, ‘Assess’, ‘Advise’, ‘Assist’, and ‘Arrange’

*PHC-SCP* Public health centre smoking cessation program, *MOGEF-SCP* Ministry of Gender Equality & Family smoking cessation program, *NHIS-SCP* National Health Insurance Service smoking cessation treatment project, *TKM* Traditional Korean Medicine, *NA* not applicable, *NRT* Nicotine Replacement Treatment

### 1st major smoking cessation program: Public Health Centre Smoking Cessation Programme (PHC-SCP)

PHC-SCP is defined as a ‘TKM smoking cessation class’ in Table 1. It can be divided into 3 phases. In 2001, the 1st phase programme started as a pilot in 9 public health centres. PHC-SCP mainly performed acupuncture treatments. The programme was targeted at both adults and teenagers. The scale of this programme had expanded, and 137 public health centres participated in PHC-SCP in 2003 (Table 1).

In 2004, a 2nd phase PHC-SCP, which provided education as well as acupuncture, was introduced. This programme involved perceiving the harms of smoking and reaching complete smoking cessation. The programme consisted of 3 sections: training for smoking cessation, acupuncture treatment, and observing the changes in smoking patterns. Table 2 presents the specific interventions. It had been TKM-Hub public health centre period from 2005 to 2012. Although the contents of the PHC-SCP were slightly different for each local public health centre, the basic structure originated from the 2004 programme. Managing withdrawal symptoms and counselling could be optionally conducted in some public health centres.

In 2013, a 3rd phase community-based, integrated health promotion programme began in South Korea [16]. As a result, non-smoking programmes using TKM alone have stopped and most have been replaced with those that are associated with non-smoking clinics in public health centres. The addition of TKM to existing non-smoking clinic programmes was expected to be synergistic as each department could be involved in the programme. For example, patients could receive advice from counsellors, acupuncture from the Korean medicine clinics and both an NRT prescription and urine test from the internal medicine clinics.

### 2nd major smoking cessation program: Ministry of Gender Equality & Family Smoking Cessation Programme (MOGEF-SCP)

The MOGEF-SCP is defined as an ‘Ear acupuncture programme for teenagers’, and was organised by the MOGEF with the MW in 2003. The purpose of this programme is to help teenagers control their tobacco habits and to reduce the smoking rate in the community at no cost for the participants. Table 1 presents basic information about this programme.

The MOGEF-SCP is divided into two subtypes, a ‘designation clinic system’ and a ‘designation doctor system’ (Table 1). The designation clinic system assigns particular TKM clinics as ear acupuncture treatment institutions, and they offer ear acupuncture to smoking teenagers. The ‘designation doctor system’ involves TKM doctors who visit schools and provide ear acupuncture. The designation doctor system began in 2010. One middle school and one high school are selected by each local organization to participate in the programme. Ear acupuncture is used as main the intervention twice a week for 3 weeks (Table 2). Health counselling and education are also carried out, and a non-smoking pill, which is a TKM smoking cessation aid, is offered as needed. The herbal compositions of the non-smoking pill are *Pueraria lobate* Ohwi*, Glycyrrhiza uralensis, Zingiber officinale, Platycodon grandiflorum, Inula helenium, Mentha piperita, Adenophora triphylla, Amomum xanthioides, Rhynchosia nulubilis, Dryobalanops aromatica, Cinnamomum cassia, Citrus unshiu*, and *Fritillaria ussuriensis* [17].

### 3rd major smoking cessation program: National Health Insurance Service Smoking Cessation treatment Project (NHIS-SCP)

The NHIS-SCP is a smoking cessation treatment services supported by the NHIS and the MW that started in 2015. The NHIS-SCP provides smoking cessation treatment to patients who want to participate up to twice a year (Table 1).

Patients visit medical institutions, such as hospitals, clinics, or public health centres, which are registered to offer smoking cessation treatment. The total treatment period is 8 or 12 weeks, and patients are provided with counselling up to 6 times and drugs or nicotine replacement therapies (NRTs) for tobacco control. A total 80% of a patient’s medical expenses are covered, and the expenses for varenicline, bupropion, and NRTs (i.e., nicotine patch, gum, and tablet) are currently covered by a pilot project budget of the NHIS (Table 2).

Table 2 shows the interventions that TKM doctors use in the process of cessation treatment. Medical consultation is included, whereas, acupuncture is not currently used in the NHIS-SCP. However, the TKM doctors can prescribe acupuncture or herbal medicines to relieve withdrawal symptoms if the patients pay for the medical expenses with their own money [18, 19]. Acupuncture for smoking cessation is provided in many TKM clinics, although it is not yet covered by health insurance. There are 1,270 registered smoking cessation treatment TKM clinics and 204 clinics carry out treatment as of March 2016 (Table 1) [20].

## Discussion

This study described smoking cessation programmes that use TKM interventions in South Korea, and the study results provide basic information for planning future smoking cessation programmes to contribute increasing smoking cessation rates and finally promoting health. In conventional western medicine, there are smoking cessation treatment guidelines for primary care physicians that were developed by the Korean Academy of Family Medicine [21]. The guidelines recommend following doctors’ advice, individual or group behavioural counselling, self-help interventions, attending smoking cessation clinics, and medicinal treatments, such as bupropion, nortriptyline, and NRT.

In China, a non-smoking clinic was established in 2007 at the Acupuncture and Moxibustion Hospital, Academy of Chinese Medical Sciences, and the patients were treated with acupuncture, massages, and herbal patches [22]. Traditional Chinese medicine (TCM) is relatively well used in cessation treatment; however, there are no national cessation programmes using TCM that are led by the government.

The PHC-SCP, also known as the TKM smoking cessation class, was included in five health promotion programmes. The budget for all 5 of the Health Promotion Programmes using Tradition Korean Medicine (HaPP-TKM) was USD 300,000 per public health centre; however, it is unclear how much was used for the PHC-SCP (Table 1). Additionally, the details of the PHC-SCP are slightly different for each public health centre. However, the PHC-SCP is integrated with western medicine programmes, and this is a strong advantage that the other programmes do not provide.

The implementation of the MOGEF-SCP is relatively easy due to the cooperation of schools, which provide good conditions for not only performing treatments but also providing education. Acupuncture is more appropriate than NRT for teenagers because NRT is not recommended for them [5]. Non-smoking pill is used in some TKM clinics optionally, according to the judgment of TKM doctors. Meanwhile, a herbal nicotine patch was developed and has been sold as general medicine in China [23].

The NHIS-SCP enhanced the accessibility to tobacco control therapy by providing medical care in clinics to anyone. Accordingly, the number of participants has overwhelmingly increased, and the budget for full support of medical costs was limited. However, this programme is still in its stages and TKM interventions, such as acupuncture and herbal medicines, are not yet covered by insurance. In Japan, cessation treatments have been covered by health insurance since 2006; however, traditional medicine is not included in the cessation treatment programmes [24].

The treatments are provided according to the guidelines for TKM cessation treatment in South Korea. According to Park’s study [25], ear acupuncture was the most frequently used TKM intervention for cessation treatment. The developed guidelines are the ‘Guideline on Acupuncture Treatment and Counselling for Smoking Cessation’ [26], developed by the Association of Korean Medicine (AKOM), and the ‘Guideline on Smoking Cessation Treatment for Health Care Providers’ [27], developed by the Ministry of Health and Welfare (MW) and the National Health Insurance Service (NHIS).

Currently, the main TKM intervention used in the smoking cessation programmes is acupuncture [25]. There are many clinical trials using ear- or body- acupuncture as an intervention in several countries [28]. As a result of the ear acupuncture treatments provided through the MOGEF-SCP, 102 of 472 (22.5%) high school students who had more than 5 ear acupuncture treatments succeeded in complete smoking cessation, and 360 (75.6%) showed smoking cessation or significant smoking reduction [29]. Herbal medicines were used to help relieve withdrawal symptoms, including anxiety, increased appetite, and phlegm.

As the study of the national lead T&CM in smoking cessation programmes was few, our study results provide details about the interventions used in TKM smoking cessation programmes in South Korea (Table 2). This is also differentiation of this study because T&CM is not included in national lead cessation programme in other countries. On the basis of our study, information can be utilised when planning smoking cessation treatments with T&CM in other strategies or countries. Furthermore, the study results suggest future research plans for tobacco control studies.

However, there are limitations of TKM smoking cessation programmes. First of all, cessation rate of each programme was not reported. It is the biggest weakness of this study that comparing endpoints of three programmes cannot be done. Second, TKM is still not highly used in smoking cessation treatment. This should be followed by government support. TKM interventions are not supported in the NHIS-SCP and the medical cost is relatively expensive compared with that of conventional western medicines. Third, the evaluation of TKM programmes is insufficient. Quantitative assessments, such as urine tests and pulmonary function tests, and qualitative assessment should be utilised to properly evaluate the programmes. In last, since the lack of reporting form and system, some information such as the number of participating institutions, and budgets were unable to report. The reporting system should be established to keep sustainable programmes. Fourth, a standard guideline for TKM smoking cessation treatment based on well-designed trial results is needed to support its efficacy and safety. Finally, various TKM interventions, such as herbal medicines and moxibustion, should be developed to increase smoking success rate.

## Conclusions

In South Korea, traditional medicine has been used for smoking cessation treatment mainly in primary medical institutions. This study summarised Korean smoking cessation programmes that use TKM, and it is the first article to provide related information and a brief history of each programme. This will be a reference for other strategies that are to apply traditional medicine to their smoking cessation programmes.

## Additional file

Additional file 1: Guideline on Health Insurance supported smoking cessation treatment. (PDF 3560 KB)

## Abbreviations

AKOM: Association of Korean Medicine
FCTC: Framework Convention on Tobacco Control
MOGEF: Ministry of Gender Equality and Family
MOGEF-SCP: Ministry of Gender Equality and Family smoking cessation program
MW: Ministry of Health & Welfare
NHIS: National Health Insurance Service
NHIS-SCP: National Health Insurance Service smoking cessation treatment project
NRT: Nicotine replacement therapy
PHC-SCP: Public health centre smoking cessation program
PHIS: Public Health Information System
T&CM: Traditional and alternative medicine
TCM: Traditional Chinese medicine
TKM: Traditional Korean medicine
WHO: World Health Organization
