# Supplementary material for: Smoking cessation programmes using traditional medicine in Korea
Source: BMC Complement Altern Med. 2016 Dec 1;16:494. doi: 10.1186/s12906-016-1462-9 (PMC5134287; doi:10.1186/s12906-016-1462-9)
Supplement: Additional file 1: — Guideline on Health Insurance supported smoking cessation treatment. (PDF 3560 KB) [file 12906_2016_1462_MOESM1_ESM.pdf]

2015

금연치료 건강보험 지원사업안내

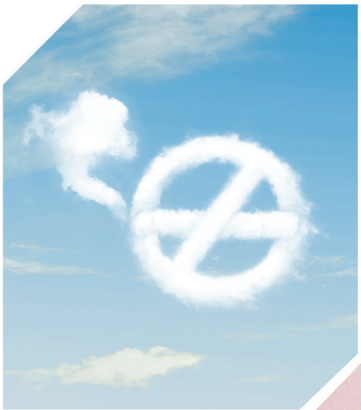

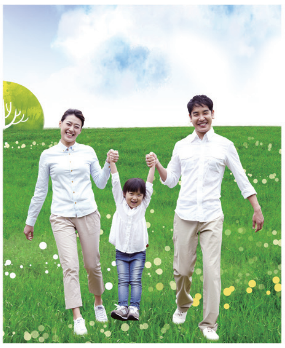

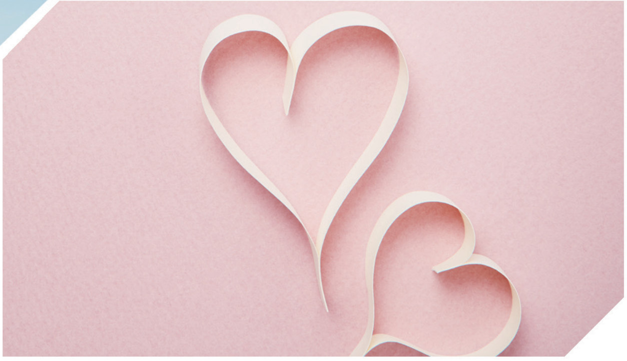

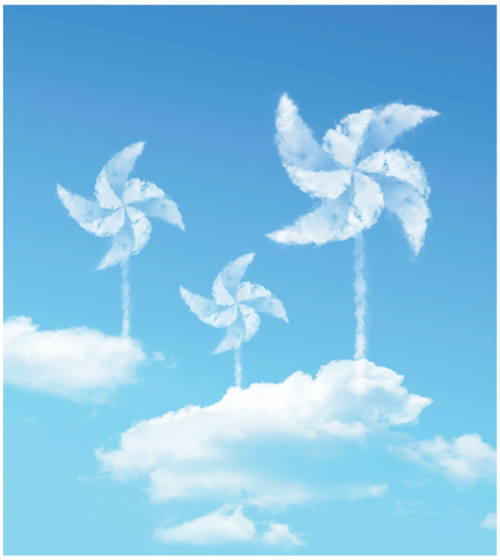

보건복지부
MINISTRY OF HEALTH & WELFARE

h·well
국민건강보험
National Health Insurance Service

[제1장]

금연치료 건강보험 및 저소득층 지원사업 지침

Ⅰ. 사업 개요

1. 사업 목적 ········· 1
2. 근거 법령 ········· 1
3. 사업 내용 ········· 1

Ⅱ. 의료기관 지원 절차 및 방법

1. 제공기관 및 의료인력, 금연참여자 등록 ··· 3
2. 지원 기준 ········· 4
3. 진료 · 상담료 ········· 4
4. 금연치료의약품 처방기간 및 방법 ········· 8
5. 의료기관 진료 · 상담료 지원 및 부담 기준 9
6. 의료기관 진료 · 상담료 신청 ········· 10

Ⅲ. 약국 지원 기준 및 절차 등

1. 금연참여자 확인 ········· 11
2. 금연치료의약품 및 금연보조제 인정 기준 11
3. 금연치료의약품 및 금연보조제 지원과 부담 ········· 11
4 금연치료의약품비 등 신청 방법 ········· 13

Ⅳ. 지원비용 지급 및 정산 등

1. 금연치료 지원 비용 지급 ········· 15
2. 금연치료 지원 비용 정산 ········· 15

Ⅴ. 인센티브 지급

1. 금연참여자 인센티브 ········· 16
2. 의료기관 인센티브 ········· 16

Ⅵ. 금연치료 사업추진 협의체

1. 사업추진 협의체 구성 ········· 17

Ⅶ. 서식 · 붙임

서식

[서식1] 금연치료 의약품 처방전 ········· 19
[서식2] 금연치료 등록(상담) 대장 ········· 20
[서식3] 금연치료 문진표 ········· 21
[서식4] 금연보조제 상담확인서 ········· 22
[서식5] 의료기관 영수증 ········· 23
[서식6] 약국 영수증 ········· 23

붙임

[붙임1] 금연보조제 목록 ········· 24

CONTENTS

[제2장]

금연치료 건강보험 및 저소득층 지원사업 전산 매뉴얼

Ⅰ. 요양기관정보마당

1. 금연치료 건강보험 지원사업 사이트 찾기 ………………… 27
2. 요양기관정보마당 로그인 방법 ……………………………… 28

Ⅱ. 금연치료관리시스템

1. 의료기관 금연치료 참여신청 등록 …………………………… 31
2. 의료기관 금연치료 상담 · 비용 입력 ………………………… 33
3. 의료기관 금연치료 상담내역 조회 …………………………… 35
4. 약국 금연치료 약제비 신청 입력 ……………………………… 37
5. 약국 금연치료 약제비 내역 조회 ……………………………… 39

*전산 프로그램 개발 과정에서 다소 변경, 수정될 수 있으며 변경 사항은 지체 없이 안내 하도록 하겠습니다.

부록

금연치료 건강보험 지원사업 FAQ …………………………………… 41

CONTENTS

[제3장]

의료인을 위한 금연진료 · 상담 안내서

Ⅰ. 흡연과 건강

1. 흡연과 사망 및 질병부담 56
2. 한국인의 흡연율 56
3. 흡연의 건강에 대한 영향 58
4. 담배규제기본협약(FCTC, Framework Convention on Tobacco Control) 59
5. 금연의 효과 60

Ⅱ. 니코틴 의존의 이해

1. 니코틴의 작용 62
2. 니코틴 의존 : 담배사용장애 (Tobacco Use Disorder) 64

Ⅲ. 금연진료의 실제

1. 금연진료의 치료 모델 66
2. 금연 전략의 적용 68
3. 정신질환이 있는 흡연자의 금연 치료 83
4. 심혈관질환이 있는 흡연자의 금연 치료 85
5. FAQ 89

제1장
금연치료 건강보험 및 저소득층 지원사업 지침

Ⅰ. 사업 개요

Ⅱ. 의료기관 지원 절차 및 방법

Ⅲ. 약국 지원 기준 및 절차 등

Ⅳ. 지원비용 지급 및 정산 등

Ⅴ. 인센티브 지급

Ⅵ. 금연치료 사업추진 협의체

Ⅶ. 서식 · 붙임

지원 업무절차 요약

의료기관 등록

금연치료 참여 신청

① 요양기관정보마당(http://medi.nhis.or.kr) 접속
② 공인인증서 로그인
③ 「금연치료관리시스템」 ➡ 「의료기관 참여 신청 관리」
④ 의료기관기호 입력 ➡ 조회
⑤ 신청인 성명, 근무부서, 사무실 전화 등 정보 입력 ➡ 저장

의료기관 금연참여자 내원

등록

① 「의료기관 금연치료 진료 · 상담 등록 관리」 클릭
② 금연참여자 주민등록번호 입력 ➡ 조회
③ 금연치료 등록 또는 진료 · 상담 이력 확인

최초 등록자	기 등록자
(서식3) 금연치료문진표 작성, 등록<br>대상자 기본정보 등록	진료

진료

① (서식2) 금연치료 등록(상담)대장 작성
② 「의료기관 금연치료 진료 · 상담 등록 관리」 화면 등록

1~5회	최종 회
• 진료일<br>• 의료기관 기호<br>• 면허번호<br>• 처방내역<br>• **차기진료일 입력**	• 진료일<br>• 의료기관 기호<br>• 면허번호<br>• 처방내역<br>• **최종 결과 입력**<br>※ (차수, 종결일자 · 방법 · 사유)

③ 차기진료일 안내
– 차기진료일로부터 1주일 이상 내원하지 않으면 1차 지원 중단
※ 중단자 2차 등록 안내

의료기관 금연참여자 내원

수납

① 금연치료 처방전 또는 상담확인서 발행

- 금연치료 처방전 또는 상담확인서 금연의약품명, 비용 확인
- 금연치료 처방전 발행 후 반드시 자격, 비용 확인

② 자격에 따른 부담액(최초상담료 15,000원, 유지상담료 9,000원)

구 분	최초상담료		금연유지상담료	
	공단 · 국고 지원	본인부담	공단 · 국고 지원	본인부담
건강보험	10,500원	4,500원	6,300원	2,700원
최저 생계비 150%이하	15,000원	없음	9,000원	없음
의료급여 수급자	15,000원	없음	9,000원	없음

③ 수납

④ 차기진료일 안내

⑤ 금연치료 처방전 또는 상담확인서 발행 후 최종 저장 버튼 클릭 → 공단지원금 신청

⑥ 금연치료 기록 관리

- 금연치료등록(상담)대장[서식2], 금연치료문진표[서식3] 보관

지급조회

① 「병의원 금연치료 진료 · 상담조회」 클릭

② 의료기관 기호, 진료일자 입력

③ 신청결과 조건(전체, 지급, 미지급) 선택 → 조회

※ 대상자, 진료일자, 신청금액, 지급일, 차기진료일 확인 가능

약국

조제 및 판매 등록 경로

① 요양기관정보마당(http://medi.nhis.or.kr) 접속
② 공인인증서 로그인
③「금연치료관리시스템」➡「금연치료의약품 등록(신청)관리」

참여자 확인

① 의료기관에서 발급 된 금연치료 처방전 또는 상담확인서 수령
②「금연치료의약품 등록(신청)관리」 클릭
③ 처방전 또는 상담확인서 발행번호 입력 ➡ 조회
④ 처방 내역 조회

입력

① 의료기관 처방전 또는 상담확인서의 금연치료의약품 및 금연보조제 입력
② ▲투약일수, ▲1일 용법, ▲정 당 단가 입력하면 금액 자동 계산
③ 참여대상자 부담금만 수납
④ 저장 버튼 클릭 ➡ 비용 신청 완료
※ 지급 신청은 조제 · 판매일로부터 7일 이내 신청

수납

① 자격별 부담액

구 분	금연약국관리료		금연치료의약품 및 금연보조제		
	공단지원	본인부담	공단지원	국고	본인부담
건강보험	1,400원	600원	공단 지원금	없음	공단 지원금 초과액
최저생계비 150% 이하	2,000원	없음	공단 지원금	국고 지원금	국고 지원 한도초과액
의료급여 수급자	2,000원	없음	없음	국고 지원금	국고 지원 한도초과액

② 본인부담금 수납

지급조회

① 지급조회 가능

Ⅰ. 사업 개요

1. 사업 목적

● 흡연자에 대한 종합적인 금연치료 지원을 통해 금연 성공률을 높이고, 흡연으로 인해 발생하는 질환 및 사망의 위험을 예방함으로써 국민 건강증진을 도모하고자 함

2. 근거 법령

● 국민건강보험법 제14조(업무 등) 제1항, 제12호, 제13호

3. 사업 내용

가. 지원 예산

● 국민건강보험공단 사업비 : 건강보험 가입자

● 국고 : 의료급여수급자, 최저생계비 150% 이하 계층(이하 “저소득층”이라 한다)

나. 지원 대상

● 금연치료 참여 신청한 병 · 의원 및 보건소, 보건지소(이하 “의료기관”이라 한다)에 내원하여 금연치료를 희망하는 모든 국민에 대해 금연치료 지원

• 건강보험 가입자, 의료급여수급자, 최저생계비 150% 이하 계층

다. 제공 기관 및 의료인력 기준

● 제공기관은 국민건강보험공단(이하 “공단”이라 한다)에 금연치료 참여 신청을 등록한 의료기관 및 약국으로 함. 다만, 약국은 참여 신청 등록을 하지 않음

● 의료인력은 의사, 치과의사, 한의사, 간호사로 함(이하 “의료인력”이라 한다)

라. 지원 프로그램 구성

● 금연치료를 희망하는 모든 국민에게 1년에 2번(차수)까지 금연치료 지원

• 12주 기간 동안 6회 이내의 상담과 금연치료의약품 또는 금연보조제(니코틴패치, 껌, 정제) 투약(구입) 비용의 일부를 지원

[그림1] 프로그램 개요

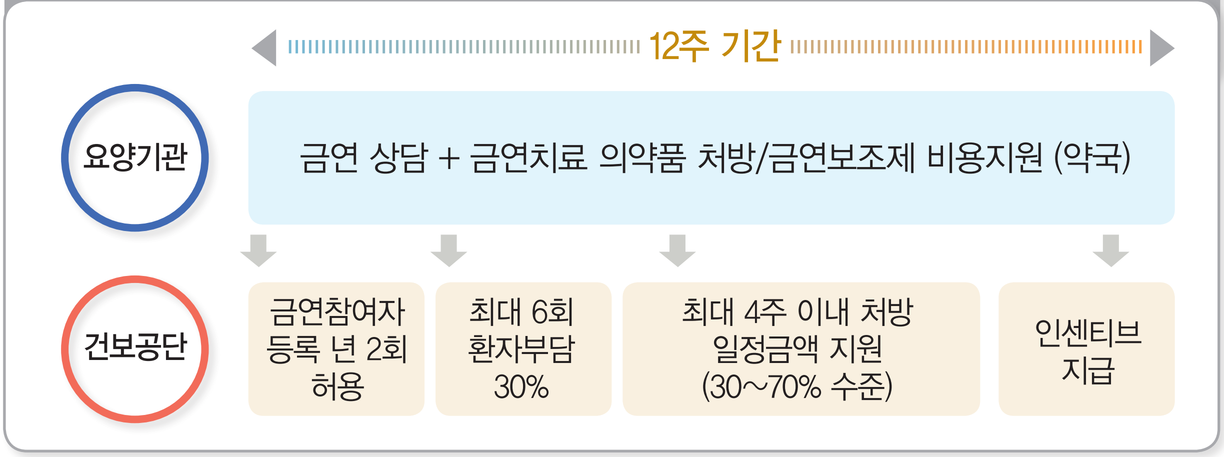

마. 지원 항목

- 의료기관 : 금연치료 진료 · 상담료
- 약국 : 금연약국관리료, 금연치료의약품비, 금연보조제비
  • 지원 성분 : 바레니클린, 부프로피온, 금연보조제(니코틴패치, 껌, 정제)

바. 지원 절차

- 금연참여자는 금연치료 의료기관에 내원하여 진료 · 상담을 받고 금연치료 의약품 처방전(이하 “처방전”이라 한다) 또는 금연보조제 상담확인서(이하 “상담확인서”라 한다)를 발급받아 약국에서 금연치료의약품 또는 금연보조제를 구입
- 의료기관은 금연참여자의 진료 · 상담료를 약국은 금연약국관리료, 금연치료의약품비 및 금연보조제 지원비를 공단으로 직접 신청

[그림2] 금연치료 건강보험 및 저소득층 지원 사업 체계

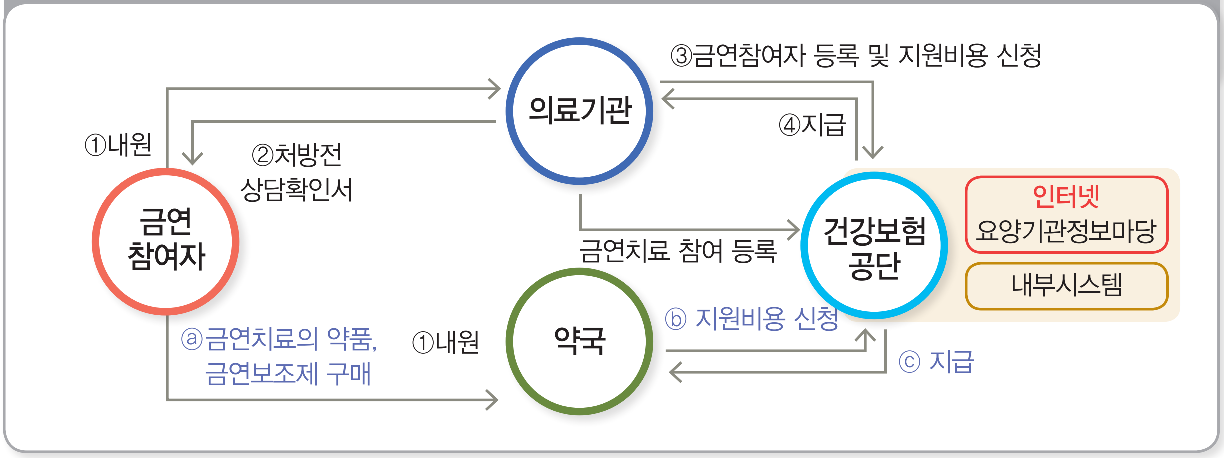

Ⅱ. 의료기관 지원 절차 및 방법

1. 제공기관 및 의료인력, 금연참여자 등록

가. 의료기관 등록

- 의료기관은 공단이 지정한 홈페이지(요양기관정보마당, http://medi.nhis.or.kr)를 통해 금연치료 참여 신청을 직접 등록. 다만, 보건소, 보건지소는 의료인력이 근무하는 경우에만 참여 신청 등록이 가능함
- 의료인력은 금연참여자에게 질높은 금연치료서비스를 제공하기 위해 금연진료관련 교육이수 필요

  ※ 교육관련 세부사항은 별도안내

나. 금연참여자 등록

- 의료기관에서는 금연참여자가 내원하여 금연치료를 요청하는 경우 공단의 「금연치료관리시스템」(이하 "전산"이라 한다)에 등록하여야 함
- 공단은 금연치료를 등록한 금연참여자 정보를 관리 함

다. 금연참여자 등록 방법

- 경로 : 공단 요양기관정보마당(http://medi.nhis.or.kr)
- 방법 : 공인인증서 로그인

  ※ 금연참여자 등록 방법 관련 세부사항은 '전산사용 설명서' 참조(전산사용 설명서는 전산개발 상황에 따라 변경될 수 있음)

2. 지원 기준

가. 지원 횟수

- 건강보험 재정 및 국고의 효율적 활용과 남용 방지를 위하여, 1년에 2번(차수)까지 지원하고, 평생지원 횟수는 향후 추가 검토
  - 첫 번째(1차) : 최초 금연시도 금연참여자
  - 두 번째(2차) : 1차수 프로그램 중단 자 또는 1차수 시도자 중 12주 초과자

    ※ '1년에 2번'은 '회계연도 내 2번' 지원을 의미함

나. 지원 내용

- 차수별 12주 동안 6회 이하의 진료 · 상담과 금연치료의약품 및 금연보조제 비용을 건강보험 사업비 및 저소득층 지원 사업비로 지원함
- 예정된 차기 진료일로부터 1주 이내에 의료기관을 내원하지 않을 경우 금연치료 프로그램 참여 중단으로 간주하여 해당 차수 지원은 종료됨

  ※ 지원종료자에 대하여 2차수 등록 안내

3. 진료 · 상담료

가. 정의

- 금연치료를 희망하여 내원한 금연참여자에게 금연치료 프로그램을 충분하게 이해를 시키고, 금연참여자가 금연을 성공할 수 있는 의학적, 심리적 제반사항을 권고하여야 함
  - 금연치료 진료 · 상담은 의료인력 대면상담을 원칙으로 함

    ※ 의사의 대면 상담 후 의사의 지도하에 간호사(간호조무사 제외) 상담 참여 허용
  - 진료 · 상담 주기는 금연참여자 형편 등을 고려하여 의료인력이 정함
  - 의료기관은 충실한 상담 제공을 위해 「금연치료 등록(상담)대장[서식2]」, 「금연치료 문진표[서식3]」을 작성(수기 또는 전산입력)하여 관리함
  - 금연진료 · 상담에 관한 사항은 제 3장, "의료인을 위한 금연진료 · 상담 안내서" 참조

나. 대상자

- 대상자 구분 : ①건강보험 ②최저생계비 150% 이하 계층 ③의료급여수급자

● 중단처리 : 예정된 차기 진료일로부터 1주 이내에 의료기관을 내원하지 않는 경우 금연참여자가 금연치료 참여를 중단(금연치료 프로그램 참여 중단)한 것으로 간주하여 공단에서 중단처리하고 해당 차수 지원을 종료함

다만, 금연참여자 본인의 이사 · 출장 · 질병입원 또는 금연치료 의료기관의 휴 · 폐업, 휴진의 경우에는 계속적인 지원이 가능

※ 의료기관에서는 예외 사유에 해당하는 금연참여자의 경우 전산에 ‘지원 중단상태’를 ‘지원 유지 상태’로 변경하여 계속 지원

(예시) 유지상태로 변경하는 방법 : 지원중단 해제 사유를 입력 후 저장

다. 진료 · 상담 유형

● 공통 사항

• 의료인력은 금연치료를 받는 금연참여자의 니코틴중독상태, 금연의지, 부작용 가능성, 금연참여자 선호도 등을 고려하여 금연치료의약품 또는 금연보조제에 대해 상담

– 금연치료의약품은 금연참여자와 상담 후 의사, 치과의사가 처방(주 단위)

– 금연보조제는 의사, 치과의사, 한의사의 상담을 받아 금연참여자가 결정

• 차기 진료일은 처방 기간 내로 정하여, 금연참여자에게 12주 동안 연속적인 지원이 이루어 질 수 있도록 함

• 금연참여자 등록 시 SMS 서비스 동의 여부를 확인 후 ☑하여 등록, 만약 금연참여자가 수신을 거부할 경우에는 “✓ 표시” 지우고 저장

※ SMS서비스 동의 시 공단에서 차기 진료일 등을 안내함

• 안내 사항

– 차기 진료일부터 1주일 이내 의료기관을 내원하지 않는 경우 금연치료 프로그램 중단으로 간주하여 첫 번째(1차) 지원이 종료 처리됨을 반드시 안내하여야함

– 1차수 금연 실패한 금연참여자 또는 중단자는 2차수 금연치료 지원 안내

– 금연참여자에게 금연치료의약품 및 금연보조제의 종류, 효능, 부작용 등 정보를 제공

● 금연참여자 상담 시 고려 사항

① 최초상담 : 금연참여자가 최초로 금연치료 받으려고 내원 한 경우

② 금연유지상담 : 금연치료 내역 등록되어 있는 금연참여자가 내원 한 경우

③ 최종 금연유지상담 : 해당 차수의 마지막 상담이 되는 경우

※ 마지막 상담 시에는 금연 성공 여부를 반드시 확인하여야 함

[그림3] 진료 · 상담 유형

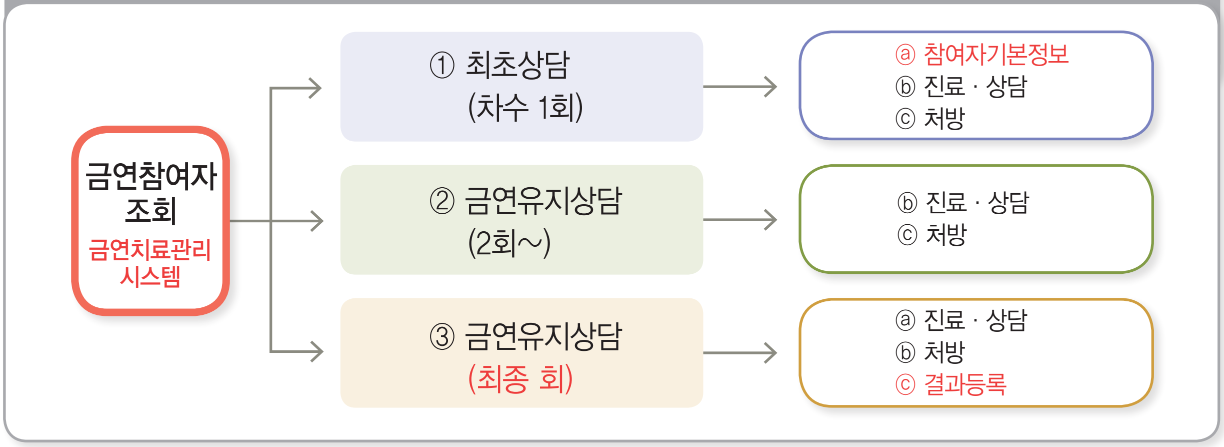

1) 최초상담(금연참여자 등록)

- 금연참여자가 내원하면 전산을 통해 금연치료 등록, 진료 · 상담 이력 확인(조회)
  - 대상자 구분 : ① 건강보험 ② 최저생계비 150% 이하 계층 ③ 의료급여수급자
    - ▷ 금연참여자 기본정보 입력 ➡ 최초진료 · 상담 입력 ➡ 처방전 또는 상담확인서 발급 ➡ 저장 ➡ 종료

최초상담 예시

- ▶ 흡연상태 : 흡연량, 기간 확인
- ▶ 흡연의 해악 : 각종질병 유발 및 사망원인, 사회적 경제부담 설명
- ▶ 담배의 실체 : 독성, 니코틴의 중독성 설명
- ▶ 치료방법 : 약물치료, 상담치료(보건소 클리닉, 금연상담전화) 안내
- ▶ 금연 의약품 안내 : 금연치료의약품 안정성, 효과성, 부작용

2) 금연유지상담 관리

- 금연참여자의 정보조회를 통해 금연치료 진행상태(유지자와 지원중단) 여부 확인(조회)
  - ▷ 진료 · 상담 내용 입력 ➡ 처방전 또는 상담확인서 발급 ➡ 저장 ➡ 종료

[그림4] 금연유지상담 절차

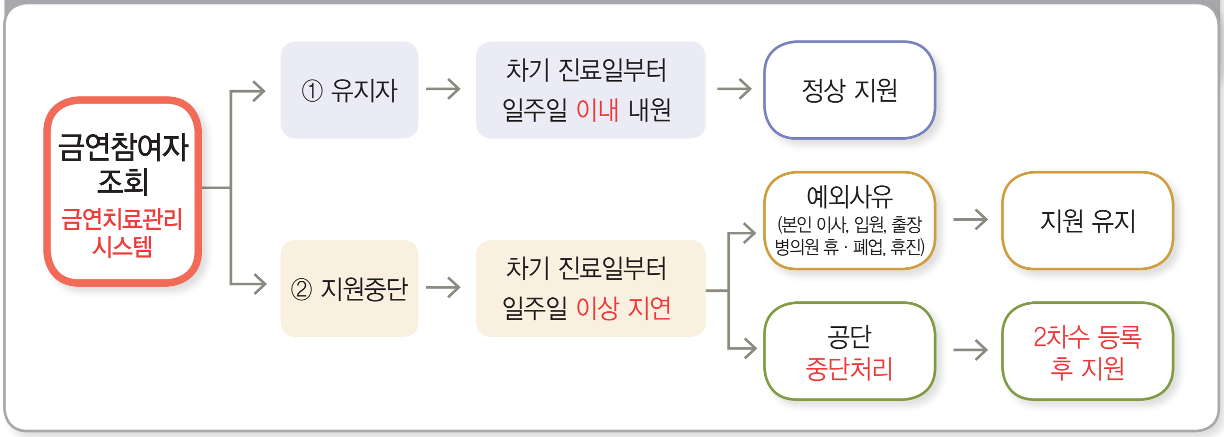

금연유지상담 예시	▶ 금연치료 경과 : 부작용, 심리상태, 스트레스, 금연의지 등 확인<br>▶ 흡연욕구 조절방법 안내 : 운동 습관화, 양치질, 차 마시기<br>▶ 금단현상 대체방법 안내 : 금연보조제(정제, 껌) 활용 등

3) 최종 금연유지상담 관리(최종결과 등록)

● 금연참여자의 금연치료 정보 조회 결과 그 차수의 마지막 회 차에 해당하는 경우
- 진료 · 상담 결과 금연에 성공한 것을 확인하였을 때
- 내원 당일의 처방에 따라 처방일수의 합이 84일(12주)이 되었을 때

▷ 최종 진료 · 상담 내용 입력 ➡ 처방전 또는 상담확인서 발급 ➡ 최종결과 입력 ➡ 저장 ➡ 종료

최종 금연유지상담 예시	▶ 금연 유지여부 확인 : 금연 성공 시 축하를 통한 격려 지지<br>▶ 흡연욕구 조절방법 안내 : 운동 습관화, 양치질, 차 마시기<br>▶ 금연 유지 방법 안내 : 금연보조제(정제, 껌) 활용, 상담치료(보건소)

라. 금연치료 처방전 및 상담확인서 발행 방법

● 금연치료 처방전 및 상담확인서는 공단의 전산에 의한 발행을 원칙으로 함
다만, 의료기관의 전산 등 장애로 전산발행이 불가능할 경우 수기에 의한 금연치료의약품 처방전 [서식1] 및 금연보조제 상담확인서[서식4]서 발행 가능함

● 금연치료 처방전 및 상담확인서 수정 또는 재발행 요구 시 등록 내용 수정 발급 가능
다만, 약국에서 금연치료의약품 및 금연보조제 조제 · 판매 등록 이후는 수정 및 재발급 불가

◆ 금연치료 처방전은 바레니클린, 부프로피온 등 전문 의약품을 처방할 때 발행
※ 한의사의 경우 금연치료의약품 처방 불가

◆ 금연치료 상담확인서는 전문 의약품 처방 없이 금연보조제만을 약국에서 구입하고자 하는 경우에 발급

마. 의료기관의 금연치료 처방전 또는 상담확인서 전산 등록

● 전산 등록은 금연치료 처방전 또는 상담확인서 발행 시 지체 없이 등록해야 하며, 다만 전산장애 등으로 불가피하게 등록이 안 되는 경우는 금연참여자가 원하는 약국에 안내

바. 의료기관 진료 · 상담 의무기록

- 의료기관은 충실한 상담 제공을 위해 '금연치료 등록(상담) 대장[서식2]' 및 '금연치료 문진표 [서식3]'를 구비하고 상담내역 기록 의무화

4. 금연치료의약품 처방기간 및 방법

가. 금연치료의약품 처방 및 금연보조제 구입에 대한 기준

- 의료기관 처방 및 금연치료 상담확인서는 1회 당 4주 이내의 범위(총 12주)

  ※ 금연 동기 유지 · 지원 등을 위해 4주 이내로 기간 제한

나. 금연치료의약품 및 금연보조제 지원 기준

- 금연치료의약품 및 금연보조제 지원은 [표1]의 1일 용법과 12주 지원 한도 내로 함
- 참여대상자에게 금연치료의약품을 처방한 경우 금연치료의약품의 효능과 효과, 부작용 등에 대한 자세한 안내를 하여야 함
- 금연치료 처방전은 금연치료의약품을 처방하고 처방전 발급 내용을 전산에 등록

[표1] 금연치료의약품 및 금연보조제 지원 기준표

구 분		금연치료의약품		금연보조제	
		부프로피온	바레니클린	니코틴패치	껌, 정제
1일 용법		2정	2정	1일 1장	1일 4~12정
12주 (84일)	공단 사업비 지원 한도	정 당 500원	정 당 1,000원	日 당 1,500원	
	국고 지원 한도액	日 당 1,360원	日 당 3,540원	日 당 2,940원	

[표2] 금연치료의약품 및 금연보조제 허가 사항

구 분	금연치료의약품		금연보조제	
	바레니클린	부프로피온	니코틴패치	껌, 정제
허 가 여 부	허가	허가	허가/신고	좌동
효능 · 효과	• 금연치료 보조<br>• 가장 좋은 효능	• 금연 단기 보조<br>• 우울증 치료제	금연 시 보조제	좌동
전 문 여 부	전문	전문	일반	일반
유 형	기타	정신신경용제	니코틴	니코틴
용 법	1일 2정 투약	1일 2정 투약	1일 1회 부착	1일 12정
투 여		최소 7주	금연의약품과 병용 안됨	니코틴패치와 병용가능
규격 종류	0.5mg / 1mg	150mg	10 / 20 / 30	2mg / 4mg

※ 금연보조제(패치, 껌, 정제)는 금연치료의약품과 병용이 불가함

5. 의료기관 진료 · 상담료 지원 및 부담 기준

가. 의료기관의 금연치료 진료 · 상담료 지원 기준

- 금연치료 건강보험 및 저소득층 지원 사업에 대한 의료기관의 진료 · 상담료 적용 기준은 [표3]과 같다.
  - 금연치료 진료 · 상담료는 최초상담료와 금연유지상담료로 구분하여 지원
  - 금연치료 진료 · 상담료는 금연참여자의 자격 유형에 의거 [표3]과 같이 부담
    - 건강보험 : 공단 사업비 70% 지원, 금연참여자가 30% 부담
    - 최저생계비150% 이하 : 공단 사업비 70% 지원, 국고 30% 부담
    - 의료급여 : 국고 100% 부담

[표3] 의료기관 금연치료 진료 · 상담료 지원 및 부담 기준표

구 분	최초상담료(1회) 15,000원			금연유지상담료(2~6회) 9,000원		
	공단지원금	본인부담	국고	공단지원금	본인부담	국고
건강보험	10,500원	4,500원	없음	6,300원	2,700원	없음
최저생계비150% 이하	10,500원	없음	4,500원	6,300원	없음	2,700원
의료급여	없음	없음	15,000원	없음	없음	9,000원

- 의료급여수급자 및 최저생계비 150% 이하 계층 국고지원 한도금액 초과액 본인부담
  - 의료기관에서 국고 부담을 공단으로 신청

[그림5] 금연치료 진료 · 상담료 신청 절차

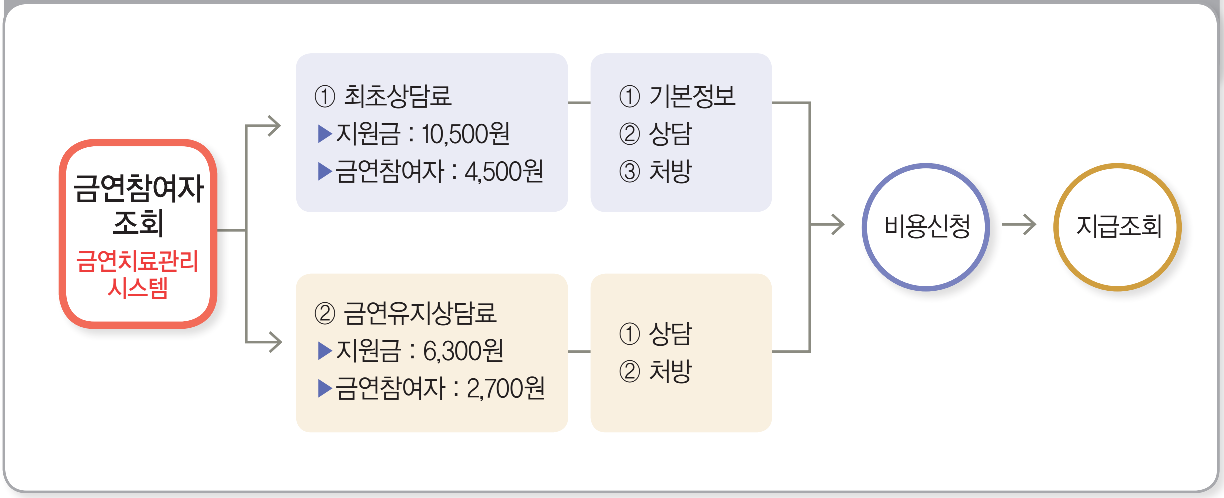

6. 의료기관 진료 · 상담료 신청

가. 신청 대상

- 금연치료 진료 · 상담만 하거나, 금연치료 처방전 또는 상담확인서를 발급한 금연참여자에 대하여 비용 신청 가능 함
- 보건소, 보건지소는 의료인력이 진료 · 상담한 경우만 신청 가능

나. 신청 방법 등

- 신청 방법 : 금연치료 진료 · 상담료는 전산에 의한 신청만 가능
- 신청 시기 : 지체 없이
  ※ 지연 등록 시 약국에서 금연치료의약품비 등 신청이 안 되므로 지체 없이 신청하여야 함

다. 진료 · 상담 신청 명세서

- Layout

참여자주민번호	자격유형	성명	자격상실일	차수	차수등록일	휴대폰번호	SMS수신	흡연기간	흡연량	체중	진료일	의료기관기호	면허번호	금연유지여부	금연성공여부	건강상태	금연치료의약품		
																	종류	투약일수	용법

차기진료일	처방전발행여부	신청액	신청일	지급일	중단코드	상담내용	최종 결과		
							종결일	종결방법	종결사유

Ⅲ. 약국 지원 기준 및 절차 등

1. 금연참여자 확인

● 금연참여자 확인
- 금연참여자가 의료기관이 발행한 금연치료 처방전 또는 상담확인서로 금연치료의약품 및 금연보조제의 조제·구입을 희망하면 약국에서는 전산을 통해 금연치료 등록한 금연참여자 여부를 확인
  - 의료기관의 금연치료 처방전 또는 상담확인서가 없이는 금연치료 건강보험 및 저소득층 지원 사업에 의한 지원이 안 됨

2. 금연치료의약품 및 금연보조제 인정 기준

● 금연치료의약품 및 금연보조제 인정 범위
- 건강보험 및 저소득층 지원 사업에서 지원하는 금연치료의약품 및 금연보조제는 식품의약품안전처에서 허가/신고 받은 품목에 한함

3. 금연치료의약품 및 금연보조제 지원과 부담

가. 금연약국관리료 지원기준

● 금연치료의약품 및 금연보조제를 조제·구입하기 위하여 금연참여자가 방문하는 약국에 대하여 금연치료 건강보험 및 저소득층 지원 사업비로 2,000원의 금연약국관리료를 지원함
- 금연참여자의 자격별 금연약국관리료 지원 및 금연참여자 부담은 [표4]에 의함

[표4] 금연약국관리료 지원 및 부담 기준표

구분	건강보험		최저생계비150% 이하			의료급여		
	공단지원금	본인부담	공단지원금	본인부담	국고	공단지원금	본인부담	국고
금연약국 관리료 지원기준	방문 당 2,000원		방문 당 2,000원			방문 당 2,000원		
	1,400원	600원	1,400원	없음	600원	없음	없음	2,000원

※ 저소득층 지원 사업은 정부에서 공단이 수탁

나. 금연치료의약품 및 금연보조제 지원과 부담 기준

● 금연치료의약품 및 금연보조제에 대한 지원 기준은 [표5]와 같다

1) 건강보험 대상자에 대한 비용 부담

– 건강보험 지원 대상의 금연참여자 부담 기준은 [표5]와 같다

[표5] 금연치료의약품, 금연보조제 지원 및 부담 기준표

구 분		금연치료 지원 사업비(건강보험)				본인부담
		부프로피온	바레니클린	니코틴패치	껌, 정제	
1일 용법		2정	2정	1일 1장	1일 4~12정	사업비 지원 한도액 초과
12주 (84일)	공단 사업비 지원 한도	정 당 500원	정 당 1,000원	日 당 1,500원		

※ 지원한도액 이내 일 경우 실 구입금액 지원

2) 저소득층 대상자에 대한 비용 부담

– 저소득층 대상 금연참여자의 지원 기준은 [표6]에 의거 [표7]과 같이 부담

[표6] 금연치료의약품, 금연보조제 지원(국고) 및 부담 기준표

구 분		금연치료 지원 사업비(저소득층)			
		부프로피온	바레니클린	니코틴패치	껌, 정제
용법		1일 2정	1일 2정	1일 1장	1일 4~12정
12주(84일)	국고 지원 한도액	日 당 1,360원	日 당 3,540원	日 당 2,940원	

※ 지원한도액 이내 일 경우 실 구입금액 지원

· 최저생계비 150% 이하 계층 : 국고 지원 한도액 초과

· 의료급여수급자 : 국고 지원 한도액 초과

[표7] 약국 금연치료의약품, 금연보조제 부담 기준표

구 분	금연치료의약품 및 금연보조제		
	본인부담	공단 지원	국고
최저생계비150% 이하	국고 지원 한도액 초과	사업비 지원 한도액	국고 지원 한도액
의료급여	국고 지원 한도액 초과	없음	국고 지원 한도액

※ 지원한도액 이내 일 경우 실 구입금액 지원

다. 약국의 금연치료의약품 및 금연보조제 비용 신청방법

● 약국은 금연치료의약품 및 금연보조제의 품목별 정 당 단가로 신청하여야 함

◆ 예시(의약품)

1정 당 단가가 1,800원인 바레니클린(챔픽스)를 2주간 처방한 경우

◆ 계산 식 : (14일 X 2정) X 1,800원 = 50,400

[표8] 지원 비용 계산 예시

(단위 : 원)

구 분		건강보험	의료급여	최저생계비 150% 이하	설명
단가		1,800	1,800	1,800	바레니클린 단가
총액		50,400	50,400	50,400	(14일 X 2정) X 1,800원
본인부담		22,400	840	840	
지원금	공단 사업비	28,000	없음	28,000	(14일 X 2정) X 1,000원
	국고	없음	49,560	21,560	14일 X 3,540원 = 49,560원

4. 금연치료의약품비 등 신청 방법

1. 신청 방법 등

● 신청 방법 : 금연치료의약품 및 금연보조제 비용은 전산에 의한 신청만 가능

● 신청 시기 : 금연치료의약품 조제일 및 금연보조제 판매일로부터 7일 이내

※ 의료기관에서 발행한 금연치료 처방전 및 상담확인서가 아니면 금연치료 지원 사업비 신청 불가

2. 금연치료의약품 및 금연보조제 비용 신청

● Layout

발행번호	주민등록번호	성명	약국기호	약사면허번호	조제판매일	조제										판매1						
						의료기관기호	의사면허번호	처방상담일	약제명	투약일	용법	정당단가	본인부담	공단부담	국고지원	종류	처방일	용법	정당단가	본인부담	공단	국고

판매2							판매3						
종류	처방일	용법	정당단가	본인부담	공단	국고	종류	처방일	용법	정당단가	본인부담	공단	국고

3. 처방전 수기 발행 건 처리

● 수기로 발행된 금연치료 처방전 및 상담확인서에 대해서 해당 의료기관의 전산장애 등으로 약국에서 조회가 안 되는 경우, 우선 금연참여자에게 조제 또는 판매하고 추후 전산 조회 확인 후 비용 신청

- 의료기관에서 금연참여자 및 금연치료 처방전, 금연치료 상담확인서를 전산에 등록해 놓지 않으면 약국에서 비용 또는 금연보조제 신청 등록 안 됨

4. 금연참여자가 의료기관의 처방전과 다른 금연치료의약품 조제를 원하는 경우

● 해당 의료기관으로부터 수정된 금연치료 처방전 발급 받은 후 가능

5. 기타

● 금연치료 처방전 및 상담확인서는 1곳의 약국에서만 조제 · 판매해야 함 (약국 중복 조제 및 판매불가)

- 의료기관 및 약국에서 공단으로 직접 신청하는 방식으로 전산 구현
- 처방전과 조제가 일치 하지 않으면 비용 지급이 되지 않도록 전산 구현됨

Ⅳ. 지원비용 지급 및 정산 등

1. 금연치료 지원 비용 지급

- 공단은 의료기관 및 약국이 신청한 금연치료 진료 · 상담, 금연치료의약품 등의 비용 신청에 대하여 확인 과정을 거처 지급결정하고, 신청 받은 날로부터 30일 이내에 의료기관 등의 등록계좌로 입금함
  - 지급결정 차수별 합산 지급
  - 공단은 비용을 지급하고 그 결과를 전산을 통해 의료기관 등이 확인할 수 있도록 하고, 이로써 지급결정 통지로 갈음함
  - 최종 지급액이 10원 미만의 끝수가 있을 때에는 그 끝수는 계산 및 지급하지 아니함(십원 미만 절사)

2. 금연치료 지원 비용 정산

- 공단은 의료기관 및 약국이 신청한 금연치료 비용에 대하여 채권압류 등으로 인한 정산 사유가 있는 경우에는 공단에서 금연치료 지원 비용과 정산할 수 있음

Ⅴ. 인센티브 지급

1. 금연참여자 인센티브

- 금연 성공 금연참여자 인센티브
  - 금연치료 프로그램을 모두 이수하고 최종 진료 시 금연유지에 성공한 금연참여자에 대한 본인 부담 일부 지원 등 인센티브 지급 예정

- 인센티브 지급제외자
  - 의료급여수급자 및 최저생계비 150% 이하 자

2. 의료기관 인센티브

- 금연치료 참여자의 프로그램 이수율과 금연성공률이 좋은 의료기관에 대한 인센티브 지급 예정

Ⅵ. 금연치료 사업추진 협의체

1. 사업추진 협의체 구성

● 구성

• 의약단체, 학계·현장 전문가, 공단 및 심평원 관계 직원 등 15인 내외로 구성

▶ (의약단체)

대한의사협회, 대한병원협회, 대한치과의사협회, 대한한의사협회, 대한약사회, 대한간호협회 각 1인

▶ (학계 등 전문가 및 소비자 단체)

대한금연학회, 송파구보건소, 한국보건의료연구원, 한국건강증진개발원, 한국소비자연맹 각 1인

▶ (공단 및 심평원 관계 직원)

공단 급여보장실장, 건강보험정책연구원 연구위원, 심평원 수가기획부장

※ 협의체 운영을 위해 간사를 두되, 간사는 공단의 금연치료지원팀장으로함

● 기능 및 역할

• 금연치료 건강보험 지원사업 주요사항 및 지원사업 참여자(참여의료기관)의 권리 구제에 관한 사항 등 심의

● 운영

• 회의는 필요시 수시 개최하며, 안건에 따라 전체 또는 일부 회의 개최하여 협의체 운영의 효율성 제고

Ⅶ. 서식 · 붙임

- [서식1] 금연치료 의약품 처방전
- [서식2] 금연치료 등록(상담) 대장
- [서식3] 금연치료 문진표
- [서식4] 금연보조제 상담확인서
- [서식5] 의료기관 영수증
- [서식6] 약국 영수증

- [붙임1] 금연보조제 목록

[서식1]

금연치료 의약품 처방전

가입자 자격 [ ] 건강보험, [ ] 차상위, [ ] 의료급여

※ [ ]에는 해당되는 곳에 "✔" 표시를 합니다.

의료기관 기호:

발급 번호			의료기관	명 칭	
금연 참여자	성 명			전화번호	( ) -
	주민등록번호	-		팩스번호	
상담 의료인의 성명		(서명 또는 날인)	면허종류		
			면허번호		제 호

금연치료의약품 종류	1회 투약량	1일 투여횟수	총 투약 주	용 법

사용기간	발급일부터 ( )일간	사용기간 내에 약국에 제출하여야 합니다.

210㎜×297㎜[일반용지 70g/㎡(재활용품)]

[서식2]

금연치료 등록(상담) 대장

가입자 자격 [ ] 건강보험, [ ] 차상위, [ ] 의료급여

등록일			등록차수	□1차, □2차		
성 명			주민등록번호		성별	□남 □여
주 소				이메일		
전화번호	휴대폰		SMS 수신	□예, □아니오	집	

❑ 금연으로 인한 상태(해당 여부 ○, ×)

구 분	상담 회차					
	1	2	3	4	5	6
흡연욕구 발생						
금단증상 발생						
부작용 경험						

❑ 상담내용

회 차	일 자	내 용
1		
2		
3		
4		
5		
6		

❑ 종결

구 분	□ 정상종결<br>□ 중간종결	중간종결 사유	□ 중간에 흡연(금연실패)<br>□ 연락두절<br>□ 질병 및 사망<br>□ 중단처리

[서식3]

금연치료 문진표

가입자 자격 [ ] 건강보험, [ ] 차상위, [ ] 의료급여

등록일			등록차수	□ 1차, □ 2차		
성 명			주 소			
전화번호	휴대폰		SMS수신	□예, □아니오	사무실	
건 강	신장(cm)		체중(kg)		복부둘레(cm)	
흡연상태	하루평균 흡연량(개비)		총 흡연 기간(년)		처음흡연 연령(세)	
질병력	□ 고혈압 □ 당뇨 □ 고지혈증 □ 기타( )				현재 복용 중인 약물 (그 이유)	
음주여부	□ 유 □ 무<br>※ 최근 1년간, 음주 유무		음주횟수	주 회		
			1회 음주량	소주 잔/ 맥주 잔		
흡 연 자 평 가	1) 지난 1년 동안 금연 시도 여부?<br>□ 예(가장오랫동안 금연시도 기간: ________개월 ___일) □ 아니오<br>2) 담배를 끊기 위해서 시도했던 방법은? (해당사항 모두 표시)<br>□ 자기 의지 □ 금연보조제(패치, 껌, 정제 등) □ 약물치료(부프로피온, 바레니클린)<br>□ 보건소 금연클리닉, 금연교실 □ 금연상담전화 □ 병원 금연클리닉(진료)<br>□ 금연침, 금연초, 심심초, 뜸 등 □ 전자담배 □ 기타 ___________<br>3) 금연에 실패한 이유는?<br>□ 본인의 의지가 약해서 □ 금단증상 때문에<br>□ 스트레스가 쌓여서 □ 주위의 유혹에 의해서<br>□ 금연 후 체중이 늘어서 □ 기타 ____________________					
니코틴 보조제 금기증여부	□ 최근 2주내 불안정 협심증 혹은 심근경색 □ 중증 부정맥<br>□ 뇌졸중 □ 장기적인 피부염(건선 등) □ 피부 알레르기<br>□ 임신 □ 수유 중 □ 없음					
니코틴의 존도검사 (Korean Version of Fagerstrom Test for Nicotine Dependence)	1. 아침에 일어나서 얼마 만에 첫 번째 담배를 피우십니까?<br>□ 5분 이내 (3점) □ 6-30분 (2점) □ 31-60분 (1점) □ 60분 이후 (0점)<br>2. 당신은 금연구역(병원, 도서관, 극장 등)에서 흡연을 참기가 어렵습니까?<br>□ 예 (1점) □ 아니오 (0점)<br>3. 하루 중 담배 맛이 가장 좋은 때는 언제입니까?<br>□ 아침 첫 담배 (1점) □ 다른 나머지 (0점)<br>4. 하루에 보통 담배를 몇 개비나 피우십니까?<br>□ 10개비 이하 (0점) □ 11-20 개비 (1점) □ 21-30 개비 (2점) □ 30개비 이상 (3점)<br>5. 아침에 일어나서 첫 몇 시간동안 하루 중 다른 시간보다 더 자주 담배를 피우십니까?<br>□ 예 (1점) □ 아니오 (0점)<br>6. 몸이 아파서 하루 종일 누워있는 날에도 담배를 피우십니까?<br>□ 예 (1점) □ 아니오 (0점)<br>총 점수 : (점)<br>◀ 니코틴 의존도 판정 ▶<br>1~3점: 니코틴의존도가 낮은 상태 , 4~6점: 니코틴의존도가 중간 상태<br>7~10점: 니코틴의존도가 높은 상태<br>* 1번과 4번은 흡연지표(Heaviness of Smoking Index, HSI)로 두 문항 합계가 4 점 이상이면 니코틴 의존도가 높다고 평가함.					

[서식4]

금연보조제 상담확인서

가입자 자격 [ ] 건강보험, [ ] 차상위, [ ] 의료급여

※ [ ]에는 해당되는 곳에 "✔" 표시를 합니다.

의료기관 기호:

상담 발급 번호			의료기관	명 칭	
금연 참여자	성 명			전화번호	( ) -
	주민등록번호	-		팩스번호	
상담 의료인의 성명		(서명 또는 날인)	면허종류		
			면허번호	제 호	
차기진료일					

상담 내용

▷ 금연보조제(패치, 껌, 정제)의 사용 방법, 금연참여자의 요구사항 등 상담

▷ 특이사항 :

*특이사항은 의료기관에서 등록한 상담(회차) 내용 출력 됨

210㎜×297㎜[일반용지 70g/㎡(재활용품)]

[서식5]

의료기관 영수증
(금연치료 진료·상담)

금 원

위 금액을 금연치료 진료·상담료로 정히 영수함

2015년 월 일

주 소 :
의료기관명:
사업자(법인) 등록번호:
대표자: 전화번호:

* 이 금연치료 영수증은 「소득세법」에 따른 의료비 영수증으로 공제신청에 사용할 수 있습니다.

[서식6]

약국 영수증
(금연치료의약품 및 금연보조제)

금 원

위 금액을 금연치료 의약품 또는 금연보조제 비용으로 정히 영수함

2015년 월 일

주 소 :
약 국 명:
사업자(법인) 등록번호:
대표자: 전화번호:

* 이 금연치료의약품 또는 금연보조제 영수증은 「소득세법」에 따른 의료비 영수증으로 공제신청에 사용할 수 있습니다.

[붙임1]

금연보조제 목록

구분		순번	제품명	주성분	제약사명	제조국	허가일자	비고
의약품		1	챔픽스정0.5mg	주석산 바레니클린	한국화이자제약㈜	수입	2007-03-30	※ 니코틴 미함유 제품
		2	챔픽스정1mg	주석산 바레니클린	한국화이자제약(주)	수입	2007-03-30	
		3	웰부트린서방정 150mg	부프로피온염산염	㈜글락소스미스클라인	수입	2002-01-18	
		4	웰부트린엑스엘정 150mg	부프로피온염산염	㈜글락소스미스클라인	수입	2007-03-02	
		5	니코피온서방정 150mg	부프로피온염산염	한미약품(주)	한국	2008-02-18	
		6	웰트론서방정 150mg	염산부프로피온	대화제약(주)	한국	2007-06-31	
		7	웰부피온서방정 150mg	염산부프로피온	슈넬생명과학(주)	한국	2007-05-31	
		8	웰서방정 150mg	부프로피온염산염	유니메드제약(주)	한국	2008-02-28	
		9	웰정(수출명 : 디쁘레오정) 100mg	부프로피온염산염	유니메드제약㈜	한국	2002-01-18	
		10	프리온서방정 150mg	염산부프로피온	㈜콜마파마	한국	2007-05-21	
보조제	패치	1	니코스탑-10패취	니코틴	㈜삼양바이오팜	한국	1997-08-27	
		2	니코스탑-20패취	니코틴	㈜삼양바이오팜	한국	1997-01-15	
		3	니코스탑-30패취	니코틴	㈜삼양바이오팜	한국	1997-08-27	
		4	니코틴엘TTS10	니코틴	한국노바티스(주)	수입	1998-04-30	
		5	니코틴엘TTS20	니코틴	한국노바티스(주)	수입	1998-04-30	
		6	니코틴엘TTS30	니코틴	한국노바티스(주)	수입	1998-04-30	
		7	니코덤7패취	니코틴	(주)한독약품	한국	1995-10-21	
		8	니코덤14패취	니코틴	(주)한독약품	한국	1995-10-21	
		9	니코덤21패취	니코틴	(주)한독약품	한국	1995-10-21	
		10	니코레트패취5mg/16hours	니코틴	한국존슨앤드존슨판매(유)	수입	2007-04-05	
		11	니코레트패취10mg/16hours	니코틴	한국존슨앤드존슨판매(유)	수입	2007-04-05	
		12	니코레트패취15mg/16hours	니코틴	한국존슨앤드존슨판매(유)	수입	2007-04-05	
		13	니코레트인비지패취	니코틴	한국존슨앤드존슨판매(유	수입	2011-07-07	
		14	니코맨패취7mg/일	니코틴	(주)새한제약	한국	2007-08-28	
		15	니코맨패취14mg/일	니코틴	(주)새한제약	한국	2007-08-28	
		16	니코맨패취 21mg/일	니코틴	(주)새한제약	한국	2007-08-28	
		17	니코패취	니코틴	(주)녹십자	한국	2006-01-13	
		18	니코앤드패취	니코틴	아이큐어(주)	한국	2007-11-20	
		19	엑소덤패취	니코틴	아이큐어㈜	한국	2010-4-30	
	껌	1	니코레트껌2mg	니코틴폴라크리렉스	한국존슨앤드존슨판매(유)	수입	2007-04-05	
		2	니코레트껌4mg	니코틴폴라크리렉스	한국존슨앤드존슨판매(유)	수입	2007-04-05	
		3	니코틴엘껌2mg	니코틴	한국노바티스(주)	수입	2006-01-18	
		4	니코틴엘껌4mg	니코틴	한국노바티스(주)	수입	2006-01-18	
		5	니코맨껌2mg	니코틴폴라크리렉스	(주)새한제약	한국	2005-08-23	
		6	니코스탑껌	니코틴폴라크리렉스	㈜삼양바이오팜	한국	2009-07-28	
	정제	1	니코틴엘로젠즈민트향트로키1mg	이주석산니코틴이수화물	한국노바티스(주)	수입	2005-10-21	
		2	니코틴엘로젠즈민트향트로키2mg	이주석산니코틴이수화물	한국노바티스(주)	수입	2005-12-01	
		3	니코스탑트로키	이주석산니코틴이수화물	㈜삼양바이오팜	한국	2005-11-17	
		4	니코맨트로키	이주석산니코틴이수화물	(주)새한제약	한국	2003-11-17	
		5	니퀴틴민트트로키 2mg	니코틴폴라크리렉스	㈜글락소스미스클라인	수입	2009-07-09	
		6	니퀴틴민트트로키 4mg	니코틴폴라크리렉스	㈜글락소스미스클라인	수입	2009-07-09	
